# Comparative study of pre- and postauricular flaps for external auditory canal defect reconstruction

**DOI:** 10.1016/j.jpra.2023.06.005

**Published:** 2023-07-01

**Authors:** Fabrizio Schonauer, Giuseppe Pezone, Annachiara Cavaliere, Francesco D'Andrea

**Affiliations:** Unit of Plastic Surgery, University Federico II, Naples, Italy, Via Sergio Pansini, 5 - 80131 Napoli

**Keywords:** Ear reconstruction, Preauricular flap, Postauricular revolving door flap, Postoncologic, External auditory canal defect

## Abstract

**Background:**

Malignancies involving the external auditory canal deserve critical evaluation due to this area's aesthetic and functional importance. Flaps can be very useful for the restoration of the external auditory canal. A variety of flaps available for the surgical treatment of external acoustic meatus defects exist, depending on the precise location and size.

**Objectives:**

Our study aimed to compare aesthetic and functional results in the postoncological reconstruction of external auditory canal defects using a preauricular flap and postauricular revolving door flap.

**Methods:**

Sixteen patients treated at our plastic surgery unit for defects involving the external auditory canal between January 2014 and December 2020 were included in the study. All defects were the result of a primary or secondary skin cancer excision. Patients were divided into two groups, one receiving the preauricular flap technique and the other the postauricular revolving door flap technique.

**Results:**

Three separate visual analog scales reported excellent scores for the two procedures, though the postauricular revolving door flap had slightly better results. Both preauricular flap and postauricular revolving door flap reconstructive techniques showed good options for external auditory canal reconstruction in postexcision skin cancer patients. From an aesthetic point of view, the revolving door flap appeared to be a more elegant surgical approach in this type of reconstruction because the scar was hidden in the postauricular sulcus.

**Conclusions:**

Reconstruction with a postauricular revolving door flap allowed for a more natural movement with no external pedicle.

**Evidence-Based Medicine (EBM) Level:**

IV

## Introduction

Although many lesions of the external ear are straightforwardly managed and can be handled with local resection and reconstruction, malignancies that involve the external auditory canal deserve critical evaluation. This area of the ear is functionally important and has an important aesthetic value because of its position, making cosmetic results of great importance.^1^ However, compared with skin cancer of other facial areas, lesions of the external auditory canal can be clinically more aggressive^2^ and, therefore, must be surgically managed with great care.

Flaps can be very useful for the restoration of the auditory canal. A variety of flaps available for the surgical treatment of external acoustic meatus defects exist depending on the precise location and size.^3^ This study aimed to compare aesthetic and functional results in postoncological external auditory canal reconstruction using pre- and postauricular flaps.

**Applied Anatomy:** The human external auditory canal is approximately 2.5 to 3.0 cm long and comprises two parts: the distal cartilaginous canal and the proximal bony canal. It extends from the concha to the middle ear, ending with the tympanic membrane. The middle ear is completely isolated from the external auditory canal by this membrane, with the proximal half of the canal formed by the tympanic bone of the temporal bone. The distal half of the external auditory canal is formed by an incomplete cartilaginous tube, covered by a very thin skin (approximately 1 mm thick), in direct apposition to the perichondrium in the presence of numerous hair follicles.

There is a complex vascular network in the periauricular area that provides various donor sites for local flaps. The posterior auricular artery arises from the external carotid artery and runs through the postauricular area, giving rise to the retro-auricular artery. The superficial temporal artery divides into at least two constant branches to the ear, known as the anterior auricular artery and the superior auricular artery. Due to the abundance of connections between arteries, flaps can be elevated in both anterograde and retrograde directions.^4^^,^^5^

## Patients and Methods

Sixteen patients treated at our plastic surgery unit for defects involving the external auditory canal between January 2014 and December 2020 were included in our study. All defects were the result of a primary or secondary skin cancer excision. Patients were staged according to the Pittsburgh classification staging system of external auditory canal tumors.^6^^,^^7^ Lesions were excised according to the existing oncological protocols.^8^^,^^9^ Patients gave written informed consent for surgical excision of the lesion and the reconstructive options presented. Two surgical techniques were employed: preauricular flap and postauricular “revolving door” flap. Patients were divided into two groups: the first group (Group A) included eight patients treated using the preauricular flap technique, and the second group (Group B) included eight patients who were reconstructed with postauricular “revolving door” flap.

**Figure 1:**
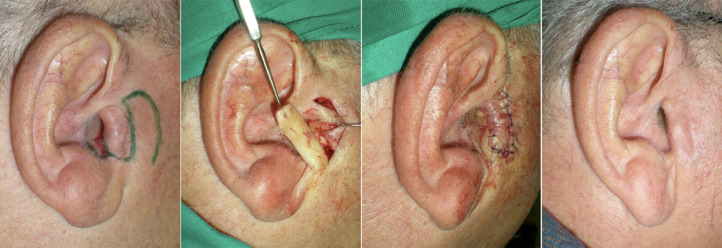
Inferiorly based pedicle preauricular flap. (a) Right ear: skin lesion occupying the external auditory canal, planned margin of excision, and preauricular flap design. (b) The auricular defect after completion of tumor excision and elevation of the preauricular flap. (c) Insetting of the flap into the defect. (d) Result at 6-month follow-up.

Markings and steps of the procedure were as follows:

**Preauricular Flap:** After resecting the lesion with oncological margins, an inferiorly based preauricular flap was outlined along the hair area in front of the ear to use the entire width of the non-hairy preauricular skin.^10^ The flap was then transposed and inset into the concha and external auditory canal area, after which the donor site was directly closed without tension^11^ (Figure 1 a, b, c, d).

**Figure 2:**
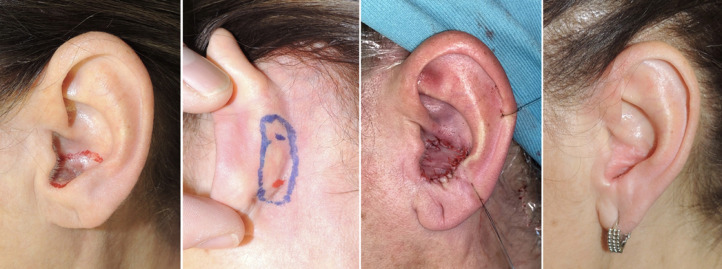
Subcutaneous pedicle posterior auricular revolving door flap. (a) Left ear: skin lesion of the auricular concha extending to the external auditory canal and planned margin of excision. (b) Posterior auricular revolving door flap design. (c) Flap transfer and skin island insetting to the anterior auricular surface. (d) Result at 6-month follow-up.

**Postauricular Revolving Door Flap**: After resecting the lesion with adequate margins, an island of the skin of the size and shape required to close the defect was outlined with a marking pen. The island was laid partly on the mastoid area and partly on the postauricular region. The proportion of the flap lying in each area varied with the position of the defect to be reached. An incision was made around this skin island, and the flap was raised, mobilizing its anterior and posterior portions. Anteriorly, the flap skin incision was integrated by creating a full-thickness “window” to move the flap from the donor area to the external auditory canal defect. The posterior skin elevation stopped at the auriculo-mastoid groove. This vertical attachment became the pedicle, i.e., the hinge point of the “revolving door.” The island was gently freed superiorly and inferiorly, leaving its central portion intact as a pedicle. This island flap was rotated like a revolving door and sutured into the defect. Lastly, the postauricular donor area was directly closed^12^^,^^13^ (Figure 2 a, b, c, d).

All the procedures were performed under local anesthetic by infiltrating mepivacaine chlorydrate with adrenaline 1:100,000. Operation time was recorded. The outcome, complications, healing time, and surgical revision procedures were recorded as well. Once a histological diagnosis of squamous cell carcinoma (SCC) was made, an ultrasound assessment of the regional lymphatic nodes was also performed. Following complete wound healing, in accordance with international follow-up guidelines for nonmelanoma skin cancer, patients with basal cell carcinoma (BCC) were clinically examined every 6 months, and patients with SCC every 3 months for the first 2 years and every 6 months thereafter for the following 3 years.^14^^,^^15^ In SCC patients, an ultrasound examination of regional lymphatic nodes was repeated once a year. After 5 years, the patients were discharged from outpatient care without any signs or symptoms of recurred or new lesions.

During the study period, at the 12-month follow-up, patients’ satisfaction with the overall outcome of the reconstructed ear was assessed using a visual analog scale (VAS) with a range between 1 and 10, where 1 denoted “very dissatisfied” and 10 indicated “impressed.” Moreover, after 12 months after surgery, a blind panel of three physicians (specialized in plastic surgery) not involved in the study evaluated the overall outcomes of both groups of patients using a VAS with a range between 1 and 10, where 1 denoted “very bad” and 10 indicated “excellent.” The use of customized VAS as an assessment scale stems from the fact that VAS is a fast and practical evaluation scale, taking less than 1 min to be filled. It is widely used due to its simplicity and adaptability to a broad range of populations and settings. In fact, it is easy to use in older patients^16^, often less alphabetized, which are the subject of this research. No training is required other than the ability to use a ruler to measure the distance to determine a score.

The following parameters were taken into consideration to define a successful outcome: the difference in skin pigmentation of the reconstructed site with the surrounding areas, the depressed contour of the reconstructed site, the donor-site scar, the presence of external auditory canal constriction, the presence of ear distortion and the overall cosmetic appearance of the external ear. Furthermore, the same panel evaluated the color and texture match of the reconstructed site with surrounding areas, giving a score between 1 and 10 in a VAS, where 1 denoted “no match” and 10 indicated “perfect match.”

## Results

Sixteen patients (10 male and 6 female) with a mean age of 72 years (range 56-88 years) were included in the study. Four patients (one in the preauricular flap group and three in the postauricular revolving door flap group) were active smokers during the operation.

Histological examinations revealed 13 BCCs, 2 BCC recurrences, and 1 SCC. The lesions were all in the external auditory canal area: 10 on the left ear and 6 on the right. All patients were classified T1 according to the Pittsburgh classification staging system of external auditory canal tumors^6^^,^^7^ (Table 1).

**Table 1:** Patient data.

Patient (N°)	Age (years)	Sex (M/F)	Ear (Right/Left)	Histology	Margins (cm)	Defect Size (cm)	Flap Type (Pre-/Postauricular)	Flap Size (cm)	Complications
1	67	M	Right	BCC	0.3	1.6 × 1.2	Preauricular	2.4 × 1.7	No
2	72	M	Left	BCC	0.3	2.3 × 1.8	Preauricular	3.4 × 2.3	No
3	80	M	Right	BCC	0.3	2.5 × 2.2	Postauricular	3 × 2.7	No
4	64	F	Left	BCC	0.3	4.1 × 3.6	Preauricular	5.4 × 4.1	No
5	85	F	Left	BCC Recurrence	0.5	3.2 × 2.1	Postauricular	3.7 × 2.6	No
6	57	M	Left	BCC	0.3	3.7 × 2.1	Preauricular	4.8 × 2.6	No
7	73	F	Right	BCC	0.3	4.2 × 2	Postauricular	4.7 × 2.5	No
8	56	M	Right	BCC	0.3	2.4 × 1.7	Preauricular	3.3 × 2.2	No
9	88	M	Left	BCC	0.3	2.4 × 3.1	Postauricular	2.9 × 3.6	No
10	67	M	Left	SCC	0.5	2.7 × 3.3	Postauricular	3.2 × 3.8	No
11	59	F	Left	BCC	0.3	3.6 × 2.7	Postauricular	4.1 × 3.2	No
12	81	M	Right	BCC	0.3	2.1 × 1.9	Postauricular	2.6 × 2.4	No
13	80	F	Left	BCC Recurrence	0.5	1.5 × 1.1	Preauricular	2.3 × 1.6	Hematoma
14	75	F	Left	BCC	0.3	3.8 × 2.6	Postauricular	4.3 × 3.1	No
15	66	M	Right	BCC	0.3	2.6 × 2.3	Preauricular	3.6 × 2.8	No
16	85	M	Left	BCC	0.3	1.3 × 1.1	Preauricular	2.2 × 1.6	No

Surgical defect size ranged from 3,68 cm^2^ to 22,14 cm^2^ (mean 9,48 cm^2^) for the preauricular flap group and from 6,24 cm^2^ to 13,33 cm^2^ (mean 10,59 cm^2^) for the postauricular revolving door flap group (*t*(14) = 3.2124, p = 0.6303).

Mean operative time was 39 min (range 29-47 min) in the preauricular flap group and 47 min (range: 37-52 min) in the postauricular revolving door flap group, with a statistically significant difference between the two groups (*t*(14) = 0.4921, p = 0.0063). Neither distal necrosis nor flap loss was observed in either group.

In one patient treated with a preauricular flap under platelet anti-aggregation therapy, hematoma occurred 12 h after the operation. Flap survived with conservative treatment.

No external auditory canal stenosis or tumor recurrence over 5 years of maximum follow-up was noted. No surgical revision was necessary for any of the patients of both groups. The mean VAS score rating patients’ satisfaction was 8,5 for the preauricular flap group and 9,0 for the postauricular revolving door flap group. No significant difference was observed between the two groups (*t*(14) = 0. 1.3333, p = 0.2037).

The mean VAS score given by physicians to overall outcomes was 8,6 for the preauricular flap group and 9,2 for the postauricular revolving door flap group. No significant difference was observed between the two groups (*t*(14) = 0. 1.9477, p = 0.0718).

The mean VAS score rating physicians’ evaluation of color and texture match was 9,0 for the preauricular flap group and the same - 9,0 - for the postauricular revolving door flap group.

## Discussion

The first preauricular flap was described by Crestinu in 1974.^10^ It was originally designed to utilize the full width of the non-hairy preauricular skin for the closure of adjacent auricular defects.^10^

The postauricular revolving door island flap was introduced by Masson in 1972 to resurface conchal defects^17^ and was also used to reconstruct defects of the scapha up to the fossa triangularis or the external auditory canal.^13^^,^^17^ The revolving door flap exploits an area of skin with a rich blood supply due to its origin and configuration, thereby minimizing the risk of necrosis.^18^ In addition, the lack of a cartilaginous substrate of the flap provides desirable elasticity without affecting the overall functional and cosmetic result. The inset of the flap takes place in a location well supported by the surrounding cartilage framework, thus preserving the natural curvature of the local auricular region without distorting the earlobe.

Posterior auricular flap versatility in partial ear reconstruction has been described.^5^ The revolving door island flap has been deemed a good option, particularly for the reconstruction of the concha and of the scapha, with excellent functional and aesthetic results.^12^

Our group research has involved different aspects of external ear reconstruction using non-surgical^19^, ^20^, ^21^, ^22^ surgical^23^, ^24^, ^25^ and microsurgical^26^ techniques.

This study observed excellent scores in the three VAS for both groups, though slightly better scores were rated for the revolving door flap patients’ group. All flaps resulted in good shape, color, and texture, with no postoperative donor-site deformities, ear canal constriction, or distortion.

Both reconstructive techniques satisfied our functional and aesthetic surgical goals, achieving appropriate and natural thickness, texture, and color for the matching skin. From an aesthetic point of view, we felt that the postauricular revolving door flap represents a more elegant surgical approach for this type of reconstruction because the scar was hidden in the postauricular sulcus. Reconstruction with a postauricular revolving door flap allowed for a more natural movement with no external pedicle. On the other side, the preauricular flap scar was visible anterior to the ear, and the pedicle bridge was sometimes evident.

In conclusion, both the preauricular and postauricular revolving door flap reconstructive techniques resulted in good options for postoncological external auditory canal reconstruction with satisfactory aesthetic and functional results.

## Conflict of Interest

The authors declare that they have no known competing financial interests or personal relationships that could have appeared to influence the work reported in this paper.
